# Management of COVID-19-Related Severe Acute Respiratory Distress Syndrome in a Patient With Active Human Immunodeficiency Virus Infection

**DOI:** 10.7759/cureus.18045

**Published:** 2021-09-17

**Authors:** Rajesh Panda, Pooja Singh, Saurabh Saigal, Sunaina T Karna

**Affiliations:** 1 Department of Anaesthesiology and Critical Care, All India Institute of Medical Sciences, Bhopal, IND

**Keywords:** human immunodeficiency virus, co-infection, sars-cov2, covid-19, acute respiratory distress syndrome

## Abstract

Our understanding of the risk of developing severe coronavirus disease 2019 (COVID-19) infection in the population living with HIV is still evolving. In this report, we discuss the successful management of COVID-19-related acute respiratory distress syndrome (ARDS) in an active case of the human immunodeficiency virus (HIV) infection; the patient had an atypical presentation characterized by very rapid progression in the severity of respiratory symptoms needing invasive mechanical ventilation. The way the case was managed broke with the custom that mechanical ventilation in patients with COVID-19-related ARDS should be deferred as much as possible since the chances of survival are minimal, especially in immunocompromised patients. Although patients with HIV infection are immunocompromised and at risk of secondary infection, young age, lack of comorbidities, and early intubation may result in a good prognosis. ARDS ventilation strategy, good infection control practices, and individualized patient care can help to wean patients off mechanical ventilation. Recent evidence does not support the use of antiretroviral drugs for prophylaxis or treatment of COVID-19 infection.

## Introduction

The coronavirus disease 2019 (COVID-19) outbreak was declared as a pandemic by the World Health Organization (WHO) on March 11, 2020 [1]. Currently, India has the second-largest number of COVID-19 cases, with close to 3.1 million people already infected [2]. Unfortunately, it also has the third-largest HIV epidemic in the world, with 2.34 million people living with HIV infection (PLHIV) in India as of 2019 [3]. We report the first case in India that involved the successful management of an active HIV-positive case with severe acute respiratory distress syndrome (ARDS) following COVID-19 pneumonia needing invasive ventilation. To date, only one other HIV-positive case with COVID-19 pneumonia needing invasive ventilation has been successfully extubated; however, in that case, the disease was controlled with an undetectable viral load and CD4 count of 604 cells/cu mm. The patient had minimal respiratory symptoms, and intubation was employed for the protection of the airway due to post-encephalopathy seizures [4]. In another case series of five HIV-positive cases with COVID-19 pneumonia, only one needed invasive ventilation but was still in ICU after 21 days till the end of the study [5].

## Case presentation

A 35-year-old male presented to the emergency of a tertiary care hospital in Central India with complaints of fever with cough and breathlessness for three days. The patient was conscious and oriented but had labored breathing with oxygen saturation of 43% on room air. Non-invasive ventilation (NIV) was initiated with 100% FiO_2_, following which his saturation improved to 90%. He was intubated and initiated on mechanical ventilation due to impending respiratory failure with the arterial blood-gas analysis showing a PaO_2_/FiO_2_ (P/F) ratio of 70. Laboratory investigations were done and a nasopharyngeal swab for COVID-19 reverse transcription-polymerase chain reaction (RT-PCR) was sent. Point-of-care ultrasonography of lungs was done, which showed B profile with basal consolidation in both lungs. The bedside chest X-ray revealed bilateral alveolar consolidation with peripheral distribution suggestive of COVID-19-related ARDS. He was started on injection piperacillin-tazobactam, injection Clexane, and injection methylprednisolone 500 mg intravenous once daily. Prone ventilation was commenced, and subsequently, his P/F ratio improved to 125.

**Table 1:** Laboratory values on the day of admission, day five, and day of discharge

Routine blood reports	Day of admission	Day 5	Day of discharge
Total leucocyte count (K/μL)	11.14	17.91	10.86
Hemoglobin (g/dL)	12.6	12.8	11.4
Total platelet count (K/µL)	325	395	518
Neutrophils (%)	93	77	68
Lymphocytes, absolute (%)	4	14	24
Neutrophil/lymphocyte ratio (N/L)	23.25	5.5	2.83
Inflammatory markers	Day of admission	Day 5	Day of discharge
Lactate dehydrogenase (IU/L)	169	613	180
Triglycerides (mg/dL)	198	190.4	198
C-reactive protein (mg/L)	54.7	86.1	5.05

Laboratory reports on day one were notable for severe lymphopenia (4%) and a very high neutrophil-to-lymphocyte ratio (NLR) of 23, suggestive of severe physiologic stress level. RT-PCR assay returned positive and the patient was started on injection remdesivir 200 mg on day one followed by 100 mg for 10 days.

The patient's young age and lack of comorbidities were pointing against the rapid progression of the disease and clinical deterioration. Thus, the HIV ELISA test was sent, which came back positive. Past history revealed that he was a known HIV-positive case and was on antiretroviral therapy (ART) for the last five months. He was on a combination regimen of dolutegravir, lamivudine, and tenofovir. CD4 counts were 177 cells/mm^3^ with a viral load of 24,000 copies/ml. Methylprednisolone was stopped, and injection dexamethasone 8 mg IV twice daily along with Septran-DS (prophylaxis for *Pneumocystis jirovecii*) was added to the treatment protocol. After three proning sessions in four days, lung compliance markedly improved. However, on day five, the patient developed sepsis and hemodynamic instability, requiring vasopressors. Blood cultures were sent and antibiotics were escalated empirically to injection meropenem 1 gm, injection colistin 3 MIU thrice daily, and injection caspofungin 50 mg once daily. Blood cultures revealed *Candida* and *Acinetobacter,* which was pan-sensitive. The tracheal culture was sterile for bacteria and *Pneumocystis jirovecii*. On day seven of illness, percutaneous tracheostomy (PCT) was done as the patient's P/F ratio was maintained at 180-200. He was weaned off sedation and put on spontaneous/continuous positive airway pressure (CPAP) mode. Subsequently, he was switched to a high-flow cannula through a tracheostomy tube. On day 11, a T-piece trial was given with minimal oxygen flow of 3 l/minute, followed by a trach-vent filter on room air. As he was maintaining oxygen saturation with adequate cough and intact sensorium, he was decannulated on day 12. Paired nasopharyngeal and throat swab for COVID-19 RT-PCR was repeated, which returned negative. The patient was thus discharged and advised to follow up after 10 days.

## Discussion

To date, it is not clear if PLHIV are at greater risk of COVID-19 than the general population [6]. Several small cohort studies in hospitalized PLHIV with COVID-19 infection have suggested comparable clinical outcomes and risk of severe acute respiratory syndrome coronavirus 2 (SARS-CoV-2) infection when compared with the general population, particularly in those with well-controlled HIV infection (on ART and with a CD4 count >200 cells/mm^3^ and suppressed viral load) [7]. The highest rate of admission to the ICU is observed among patients aged between 60-79 years (60%), and the same age group has also been associated with the highest rates of invasive mechanical ventilation [8].

The presentation of the current case was atypical as the patient was of a relatively young age (35 years), and he presented with severe ARDS needing invasive ventilation within three days from the onset of symptoms. With such atypical presentations, HIV could be a risk factor for the rapid progression of the disease in the absence of any other risk factors like advanced age and comorbidities. Since the patient was intubated relatively earlier in the course of the disease and was ventilated under sedation, there was no self-inflicted lung injury (P-SILI), which prevented any parenchymal damage that may have otherwise occurred due to labored breathing pattern at presentation. Early PCT helped him to maintain ventilator synchrony without the need for any sedation. Further, the lack of opportunistic infections due to continued ART leading to low viral counts might have helped in decreasing the severity of the disease and early recovery of the patient.

WHO does not recommend the use of antiretrovirals either as a treatment or preventive method for COVID-19. Both the Recovery Trial and WHO multicounty adaptative trial (Solidarity Trial) showed that lopinavir/ritonavir (LPV/r) does not contribute to any reduction in mortality, duration of hospital stay, and risk of progressing to invasive mechanical ventilation or death [9,10].

## Conclusions

Based on our findings, immunocompromised populations such as HIV patients with COVID-19 co-infection can have very atypical presentations with a very rapid progression of the disease. Young age, lack of comorbidities, early hospital admission, and the initiation of invasive ventilation may result in a less turbulent course and better prognosis in this subset. Lung-protective ARDS ventilation strategy, good infection control practices, and tailored patient care can help us in managing these patients successfully. Recent evidence suggests no role for ART in the prophylaxis and treatment of COVID-19 infection.
